# Supplementary material for: Patterns of Intron Gain and Loss in Fungi
Source: PLoS Biol. 2004 Nov 30;2(12):e422. doi: 10.1371/journal.pbio.0020422 (PMC532390; doi:10.1371/journal.pbio.0020422)
Supplement: Table S1 — Also available at http://genes.mit.edu/NielsenEtAl/. (4.3 MB ZIP). [file pbio.0020422.st001.zip › NielsenEtAl/html/457.html]

AN1699.1.NCU02287.1.MG03418.1.FG10790.1


```
 CLUSTAL W (1.82) Multiple Sequence Alignments - Introns Inserted


Sequence 1: MG03418.1	540 aa
Sequence 2: FG10790.1	562 aa
Sequence 3: NCU02287.1	546 aa
Sequence 4: AN1699.1	564 aa
Alignment Length: 569 aa
Number Identitical Residues: 372 aa
Alignment Score (without introns) 16638


MG03418.1 	MSKVFTTSDVASHNKPNDLYIIVDGDVYDLTKFQDDHP-~---G~GKKI~LTRVAGKDAS
NCU02287.1	MSKIFTQADVSSHSKPDSLWIVIDGDVYDVTKFADDHP-~---G1GKKI~LQRVGGKDAS
FG10790.1 	MSKTFTTSDVASHNKPGDLYIIVDGDVYDLTKFQDDHP-~---G1GKKI~LQRVAGKDAS
AN1699.1  	MAKTFSKEDVAKNNKPDSLWIIVDEDVYDLTKFQDEHPA1PRIG~GKKI1LTRVAGKDAS
          	*:* *:  **:.:.**..*:*::* ****:*** *:**: .  * **** * **.*****

MG03418.1 	KAFWKYHNEGILKKYQSKLQVGSLDTK-KAAAP--APAPAAVVKK--EPAKPIVRKN-NE
NCU02287.1	KQFWKYHNEGILKKYQGKLQIGSLDTKPKAAAPAPAAAPAPAPKAAPKPKASTASSSSHE
FG10790.1 	KQFWKYHNEGILKKYKNQLLVGSLDSKPKAPEPKAAPVPAALTPKTVKTVEKEVAAK-EE
AN1699.1  	KQFWKYHNEGILKKYKSQLQIGSLDSKKAPEAPAAAATEAPKKPQAAQPVDVSSAQS---
          	* *************:.:* :****:*  .  * .*.. *.    : :.       .   

MG03418.1 	ESEALDPFGEMIPFGDPGWYQS0YHSPYFNESHAALRAEVREWVQTAIEPNVTEWDEKKM
NCU02287.1	QSEALEPFGQLIPFADPSWYQS0YHSPYYNETHAALRAEIREWVETAIDPYVTEWDEKKE
FG10790.1 	EAEALEPFGDQIPFADPAWYQS0YHSPYFNESHAALRAEIREWIENDIEPHVTDWDEAKE
AN1699.1  	-AGPQDPYGDLIPFADPSWYQG0YHSPYFNQTHAALRAEVREWVEKEIEPYVTEWDEAKE
          	 : . :*:*: ***.**.***. *****:*::*******:***::. *:* **:*** * 

MG03418.1 	CPPEIYKEMGRRGYLAGLLGTKYQSKYVDN-PIKCVAEQDWDLFHEMLITDELSRAGSGG
NCU02287.1	VPAEIYKEMGKRGYLAGLLGTKYQSNYVEN-PIKSVPAEKWDLFHEMLVTDELSRTGSGG
FG10790.1 	VPEEIYKEMGRRGYLAGLLGVKYPTQYAPEGGVKSVPAEKWDLFHEMIITDELSRTGSGG
AN1699.1  	VPAKIYKQMGERGYLAGLLGVKYPTQYTPH-RVQSVAPENWDLFHEMLLTDELSRAGSGG
          	 * :***:**.*********.** ::*. .  ::.*. :.*******::******:****

MG03418.1 	FVWNVIGGFGIGCPPLLKFGKKALVDRIMPGILNGDKRICLAITEPDAGSDVANLTCEAK
NCU02287.1	FVWNVIGGFGIGCPPLVKFGKKPLVDRILPGILNGDKRICLAITEPDAGSDVANLTCEAK
FG10790.1 	FVWNIIGGFGIGCPPLMKFGKKALKDRIMPGILSGEKRICLAITEPDAGSDVANLTCEAK
AN1699.1  	LVWNLIGGYGIGCPPLVKYGKKALVDRILPGILAGDKRICLAITEPDAGSDVANLTCEAK
          	:***:***:*******:*:***.* ***:**** *:************************

MG03418.1 	LSEDGKHYIVNGEKKWITNGIWCDYFTTAVRTGGPGMGGVSLLLIERDFGGVTTRRMDCQ
NCU02287.1	LTEDGKHYIVNGEKKWITNGIWSDYFTTAVRTGGPGMNGVSLLLIERDFPGVSTRRMDCQ
FG10790.1 	LSDDGKHYIVNGEKKWITNGIWSDYFTVAVRTGEAGMNGVSLLLIERSEG-VSTRRMPCQ
AN1699.1  	LSEDGKHYIVNGEKKWITNGVWSDYFTTAVRTGGPGMNGISVLLIEREAGGVSTRRMDCQ
          	*::*****************:*.****.***** .**.*:*:*****.  .*:**** **

MG03418.1 	GVWSSGTTYVTFEDVKVPVENLLGKENKGFP------------------CIMTNFNHERI
NCU02287.1	GVWSSGTTYITFEDVKVPVENLIGKENQGFR------------------VIMTNFNHERI
FG10790.1 	GVLSSGTTYITFEDVKVPVENLLGKENQGFKGKFCY-IRMSQDQANMLSVIMTNFNHERM
AN1699.1  	GVWSSGTTYVTFEDVKVPVENLIGKENQGFKGKSSQPYFVVASVLTEYLVIMTNFNHERI
          	** ******:************:****:** .. . .     .  .    *********:

MG03418.1 	GIIIQCVRFSRVCFEESVKYANKRRTFGKRLIEHPVIRYKLAHMARQIEATYNWLESVVY
NCU02287.1	GIIIQCLRFSRVCYEESVKYANKRRTFGKKLIEHPVIRLKLAHMARQIEASYNWLENLIY
FG10790.1 	GIIIQSLRFSRVCYEESVKYGSKRRTFGKKLIDHPVIRMKLAHMARQIEASYNWLENLIY
AN1699.1  	GIIIQCLRFSRVCYEESMKYAHKRRTFGQKLVNHPVIRMKLAHMARQIEASYNWLENIIF
          	*****.:******:***:**. ******::*::***** ***********:*****.:::

MG03418.1 	QCEKMDDTQAMMLLGGPIAGLKAQATVTFEFCAREASQIFGGLSYSRGGQGAKVERLYRD
NCU02287.1	QCEKMGETEAMLRLGGAIASLKAQATVTFEFCAREASQIFGGLSYSRGGQGGKVERLYRD
FG10790.1 	QCERMGETEAMLRLGGPIAGLKAQSTLTFEFCAREASQIFGGLSYSRGGQGAKVERLYRD
AN1699.1  	QCQSMEETEAMLKLGGAIAGLKAQSTTTFEYCAREASQIFGGLSYSRGGQGGKIERLYRD
          	**: * :*:**: ***.**.****:* ***:********************.*:******

MG03418.1 	VRAYAIPGGSEEIMLDLSIRQSMRVAKMMGMKL
NCU02287.1	VRAYAIPGGSEEIMLDLSIRQSLRVAKMTGMKL
FG10790.1 	VRAYAIPGGSEEIMLDLSMRQSLRVAKAVGMKL
AN1699.1  	VRAYAIPGGSEEIMLDLSMRQSLRVHQMFGMKL
          	******************:***:** :  ****
```
